# Dataset of *De Novo* hybrid berry transcriptome profiling and characterization of *Piper* species (*Piper nigrum* and *Piper longum*) using Illumina and Nanopore sequencing

**DOI:** 10.1016/j.dib.2022.108261

**Published:** 2022-05-11

**Authors:** Johnson K. George, M.A. Fayad, S. Shelvy, T.E. Sheeja, Santhosh J. Eapen, Sona Charles, Anil Rai, Dinesh Kumar

**Affiliations:** a ICAR- Indian Institute of Spices Research, Kozhikode, Kerala, 673 012, India; b ICAR – Indian Agricultural Statistical Research Institute, Library Ave, Pusa, New Delhi, Delhi, 110012, India

**Keywords:** Hybrid transcriptome, Illumina, Nanopore, *Piper nigrum*, *Piper longum*, Piperine

## Abstract

*Piper nigrum* and *Piper longum* are the most popular and economically essential spice crops globally valued for their aromatic alkaloids, especially Piperine. However, Piperine synthesis pathway mechanisms are not yet well known. This work was aimed to generate the full-length comparative berry transcriptome analysis dataset of *P. nigrum* and *P. longum* by Illumina and Nanopore sequencing platforms. While short-read sequencing technology is widely using to capture transcriptome profiles, there are still some limitations due to the read length. We used Oxford Nanopore technology for long reads and the Illumina sequencing platform for short reads to generate a hybrid transcriptome assembly from half matured and fully matured berries of *P. nigrum* and *P. longum*. From *P. nigrum* and *P. longum* 37.3 million and 38.1 million raw reads were generated respectively. A total of 308369 contigs from *P. nigrum* and 267715 contigs from *P. longum* were obtained and successfully annotated. The transcriptome data revealed gene families involved in piperine and other secondary metabolite biosynthetic pathways. The raw data were uploaded to NCBI database. This dataset shed light on the further exploration of the piperine biosynthetic pathway, its transcriptomic changes, and evolution. Data generated has been submitted to SRA of NCBI with Bio samples accession: (SAMN13981803, SAMN22826456).

## Specifications Table

**Table:** 

Subject	Bioinformatics
Specific subject area	*De Novo* hybrid transcriptomics
Type of data	Table, text file, figure
How data were acquired	Illumina Hiseq^TM^4000, Oxford Nanopore Sequencing, Albacore, FastQC, Cutadapt, Porechop, Velvet, Oases and IDP-denovo
Data format	Raw (FASTQ) Analyzed Filtered
Description of data collection	The short read assembly was performed with Illumina data using Velvet and Oases, and short read assembly along with Nanopore and Illumina reads were used for the *de-novo* hybrid transcriptome assembly using IDP-*de novo*. A total of 308369 contigs from *P. nigrum* and 267715 contigs from *P. longum* were generated. The hybrid transcriptome assembly, Isoform detection and gene prediction are also processed in the analysis.
Data source location	Institution: ICAR- Indian Institute of Spices Research, Kozhikode, Kerala, India City/Town/Region: Kozhikode Country: India Latitude and longitude (and GPS coordinates) for collected samples/data: (11.298397, 75.840422), 11° 17′ 54.2292″ N, 75° 50′ 25.5192″ E
Data accessibility	Repository name: NCBI Database Bio Sample Accession No.: SAMN13981803, SAMN22826456 Direct URL to data: https://www.ncbi.nlm.nih.gov/sra/?term=11708092, https://www.ncbi.nlm.nih.gov/sra/?term=17790578 Full dataset: https://data.mendeley.com/datasets/vyr4r7mxj8/draft?a=a8fd91d3-2868-4ae5-bf3d-d6ab370ab792
Related research article	• Kokkat JG, Shelvy S, Fayad AM, et al. *In silico* assisted identification of peppery aroma compound ‘rotundone’ backbone genes from black pepper [published online ahead of print, 2021 Feb 10]. J Biomol Struct Dyn. 2021;1-7. doi:10.1080/07391102.2021.1883113 • George, J.K., Shelvy, S., Fayad, A.M. et al. De novo transcriptome sequencing assisted identification of terpene synthases from black pepper (*Piper nigrum*) berry. Physiol Mol Biol Plants 27, 1153–1161 (2021). https://doi.org/10.1007/s12298-021-00986-4

## Value of the Data

• This data includes downstream analysis such as relative abundance, differential expression, pathway analysis, and orthology relationships.
• The full-length hybrid berry transcriptome data and associated annotation of *Piper nigrum* and *Piper longum* will help to explore the molecular mechanism of the piperine biosynthetic pathway and also other important metabolites unique to each species
• The hybrid transcriptome sequences will serve as a future reference. They would be valuable resources to examine molecular characteristics of genes that play a role in the biosynthesis of beneficial secondary metabolites in both plants.
• Meta-analysis of the raw sequencing data may be carried out for further *in silico* comparative genomics studies.

## 1 Data Description

**Fig. 1:**
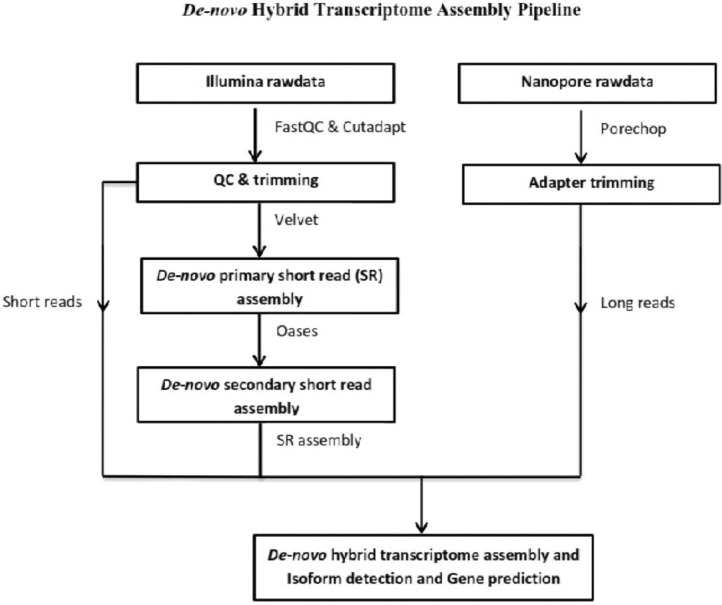
Workflow illustrating the hybrid transcriptome analysis of *Piper nigrum* and *Piper longum samples*.

Statistical report and other details of transcripts and unigenes for the full-length berry transcriptome are provided in Table 1. A total of 308369 unigenes from *P. nigrum* and 267715 unigenes from *P. longum* with an average length of 1120 were obtained. Using KOG, NR, KEGG, Pfam databases, all the unigenes were successfully annotated (Table 2). The workflow of hybrid transcriptome analysis of *Piper nigrum* and *Piper longum* samples are provided in Fig. 1. The raw reads were submitted in the NCBI database and is publicly accessible at bio sample accession no: SAMN13981803, SAMN22826456. The annotation and analysis file including the data of filtered pathways, detected SSR's, KOG, NR, KEGG and plant transcription factors are submitted in Mendeley database (https://data.mendeley.com/datasets/vyr4r7mxj8/draft?a=a8fd91d3-2868-4ae5-bf3d-d6ab370ab792).

**Table 1:** Assembly statistics of hybrid transcriptome assembly.

*De novo* Hybrid Transcriptome Assembly Statistics		
Samples	*P. longum*	*P. nigrum*
Scaffolds generated	267715	308369
Maximum Scaffold Length (bp)	16265	27769
Minimum Scaffold length (bp)	100	89
Average Scaffold Length (bp)	1120	1119
Median Scaffold Length (bp)	1027	1730
Total Scaffolds Length (bp)	299855131	345060046
Total Number of Non-ATGC Characters	85981	214451
Percentage of Non-ATGC Characters	0.029	0.062
Scaffolds >= 100 bp	267715	308365
Scaffolds >= 200 bp	252117	271562
Scaffolds >= 500 bp	193878	197948
Scaffolds >= 1 Kbp	104339	119118
Scaffolds >= 10 Kbp	106	214

**Table 2:** Statistics of annotation and analysis.

Total KOG Annotation	2189
Total NR Annotation	115173
No. of filtered pathways	2189
Total number of identified SSRs	59523
Total Pfam Domains	130720
Total KAAS - KEGG Pathway Annotation	105389
Differential gene expression (Cytochrome_P450)	476
Transcription Factors	22447

## 2 Experimental Design, Materials and Methods

1. Sample collection

Half matured and fully matured berry samples of *P. nigrum* variety IISR-Thevam and *P. longum* were collected from ICAR-Indian Institute of Spices Research, Experimental Farm, Kozhikode, Kerala, India

2. Total RNA Extraction

The modified Spectrum Plant Total RNA Kit (STRN50-Sigma) protocol was used to extract the total RNA. Bioanalyzer 2100 (Agilent, USA) was used to assess RNA integrity. An equivalent amount of RNA was pooled from three different berry samples from three different plants. Sequencing was performed on samples with an RNA integrity score of at least six.

3. Transcriptome sequencing and De novo assembly

Illumina and Nanopore sequencing were used to perform *de novo* transcriptome sequencing of berry samples. The Illumina data were demultiplexed using bcl2fastq, and nanopore fast5 data were base-called using Albacore [1]. The quality of the Illumina data was analyzed using FastQC [2]. The short reads were processed using velvet (ver. 1.1.04-ver. 0.1.21) denovo assembly pipeline[3] with the minimum kmer length was set to 69 (-hash_length 69) and maximum kmer value set to 194 (-MAXKMERLENGTH 194). Option selected for short paired read type (-shortPaired), set up two separate files for paired reads (-separate) and track the short read positions in assembly (-read_trkg yes). Data from both Illumina and Nanopore platforms and a short-read transcriptome assembly were submitted to a hybrid transcriptome assembly using IDP-denovo Assembler [4] by the parameter of kmer length 69 (-K_MER_LENGTH 69). Choose the option for left mate short reads (-SR_left), right mate short reads (-SR_right) and long nanopore reads file (-long_reads). The assembler was run with multiple threads. The *de novo* hybrid transcriptome assembly pipeline is illustrated in Fig. 1.

4. Functional Annotation

The full-length contigs were annotated by homology searches using the NR database [5] with an e-value of 1e−5. Functional annotation was performed using KOG [6] and GO [7]. The KEGG [8] parameters -species ko; E-value 1e-5 was used to compare and annotate transcripts. Contigs were classified using Pfam [9].

## Ethics Statement

Nil.

## Credit Author Statement

**Johnson K. George:** Conceptualization, Methodology, Software, Funding acquisition; **M.A. Fayad:** Visualization, Investigation, Writing- Reviewing and Editing, Software, Validation; **S. Shelvy** Validation, Investigation, Visualization, Writing- Reviewing and Editing, Software, Validation; **T.E. Sheeja:** Visualization, Investigation, Data curation, Writing- Original draft preparation; **Santhosh J. Eapen:** Supervision, Data curation, Visualization, Investigation; **Sona Charles:** Visualization, Investigation, Data curation, Writing- Original draft preparation, Software, Validation; **Anil Rai:** Visualization, Software; **Dinesh Kumar:** Visualization, Software.

## Declaration of Competing Interest

The authors declare that they have no known competing financial interests or personal relationships which have or could be perceived to have influenced the work reported in this article.

## Data Availability

Dataset of De Novo Hybrid Berry Transcriptome Profiling and Characterization of Piper sps. (Piper nigrum and Piper longum) using Illumina and Nanopore Sequencing (Original data) (Mendeley Data).
